# FlyBase: updates to the *Drosophila melanogaster* knowledge base

**DOI:** 10.1093/nar/gkaa1026

**Published:** 2020-11-21

**Authors:** Aoife Larkin, Steven J Marygold, Giulia Antonazzo, Helen Attrill, Gilberto dos Santos, Phani V Garapati, Joshua L Goodman, L Sian Gramates, Gillian Millburn, Victor B Strelets, Christopher J Tabone, Jim Thurmond, FlyBase Consortium, Norbert Perrimon, Susan Russo Gelbart, Julie Agapite, Kris Broll, Madeline Crosby, Gilberto dos Santos, Kathleen Falls, L Sian Gramates, Victoria Jenkins, Ian Longden, Beverley Matthews, Carol Sutherland, Christopher J Tabone, Pinglei Zhou, Mark Zytkovicz, Nick Brown, Giulia Antonazzo, Helen Attrill, Phani Garapati, Aoife Larkin, Steven Marygold, Alex McLachlan, Gillian Millburn, Clare Pilgrim, Arzu Ozturk-Colak, Vitor Trovisco, Thomas Kaufman, Brian Calvi, Josh Goodman, Victor Strelets, Jim Thurmond, Richard Cripps, TyAnna Lovato

**Affiliations:** Department of Physiology, Development and Neuroscience, University of Cambridge, Downing Street, Cambridge CB2 3DY, UK; Department of Physiology, Development and Neuroscience, University of Cambridge, Downing Street, Cambridge CB2 3DY, UK; Department of Physiology, Development and Neuroscience, University of Cambridge, Downing Street, Cambridge CB2 3DY, UK; Department of Physiology, Development and Neuroscience, University of Cambridge, Downing Street, Cambridge CB2 3DY, UK; The Biological Laboratories, Harvard University, 16 Divinity Avenue, Cambridge, MA 02138, USA; Department of Physiology, Development and Neuroscience, University of Cambridge, Downing Street, Cambridge CB2 3DY, UK; Department of Biology, Indiana University, Bloomington, IN 47405, USA; The Biological Laboratories, Harvard University, 16 Divinity Avenue, Cambridge, MA 02138, USA; Department of Physiology, Development and Neuroscience, University of Cambridge, Downing Street, Cambridge CB2 3DY, UK; Department of Biology, Indiana University, Bloomington, IN 47405, USA; The Biological Laboratories, Harvard University, 16 Divinity Avenue, Cambridge, MA 02138, USA; Department of Biology, Indiana University, Bloomington, IN 47405, USA

## Abstract

FlyBase (flybase.org) is an essential online database for researchers using *Drosophila melanogaster* as a model organism, facilitating access to a diverse array of information that includes genetic, molecular, genomic and reagent resources. Here, we describe the introduction of several new features at FlyBase, including Pathway Reports, paralog information, disease models based on orthology, customizable tables within reports and overview displays (‘ribbons’) of expression and disease data. We also describe a variety of recent important updates, including incorporation of a developmental proteome, upgrades to the GAL4 search tab, additional Experimental Tool Reports, migration to JBrowse for genome browsing and improvements to batch queries/downloads and the Fast-Track Your Paper tool.

## INTRODUCTION

FlyBase (flybase.org) is the leading database and web portal for data related to the fruit fly, *Drosophila melanogaster*. FlyBase contains a wide variety of data types curated from published scientific literature and incorporated from other databases, and strives to continually improve display and functionality for users. Gene Report pages have a number of sections that may be of interest to the user; where applicable, these include: genomic information, Gene Ontology (GO, 1) annotations, transcripts, protein domains, expression, alleles and transgenic constructs, phenotypes, orthologs, human disease models, physical and genetic interactions, stocks and reagents, and associated references. New users of FlyBase may find it useful to refer to Marygold *et al* (2).

In this paper, we highlight a variety of new features and improvements that have been integrated into FlyBase since our last review (3). We have introduced a number of new features that allow users to more easily find and use the data in which they are interested; these include customizable tables, improved searching using expanded Experimental Tool Reports, more links to and from other resources, and better provision of precomputed bulk files. We have upgraded tools such as Fast-Track Your Paper and ID validator. We have also introduced more high-level groupings to improve data accessibility; these include our curated Pathway Reports that facilitate understanding and exploration of a number of major signaling pathways, as well as new summary ‘ribbons’ representing expression and disease model data. In order to further promote and simplify user-led investigation of genes, we have integrated new paralog information, reviewed and revised functional data for enzymes and introduced a novel orthology-based pipeline to identify *D. melanogaster* genes potentially relevant to human disease. Finally, we give details about our genome browser change from GBrowse to JBrowse (4).

## IMPROVEMENTS FOR ACCESSING AND SORTING REAGENTS

### New responsive tables

**Figure 1.**
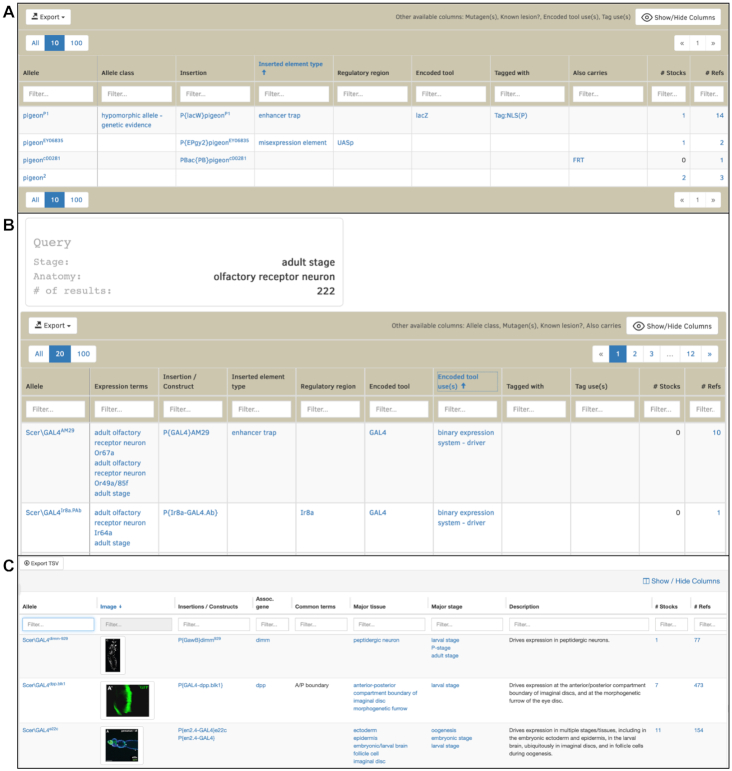
Responsive tables can be filtered, sorted and rearranged by users to easily browse or search genetic reagents. FlyBase has incorporated responsive tables into (**A**) the ‘Alleles, Insertions and Transgenic Constructs’ section of Gene Reports, (**B**) the ‘GAL4 etc’ QuickSearch hitlist and (**C**) the Frequently Used GAL4 Drivers table.

We have introduced responsive tables into our reports, allowing users to customize the display and easily access the subset of the data that is important to them. These tables are currently implemented in the ‘Alleles, Insertions, and Transgenic Constructs’ of Gene Reports, the ‘Members’ section of Pathway Reports and the hitlist of results of the QuickSearch GAL4 etc. tab (Figure 1). We plan to expand them to other tables on the website. Key features include the ability to Show/Hide columns, column reordering, row filtering and sorting. As well as providing direct links to the selected data subsets, the user-specified table can be downloaded in a number of formats and the filtered results can be exported to a FlyBase hitlist for further filtering or analysis.

### Additional Experimental Tool Reports

We continue to link newly generated transgenic alleles and constructs to any relevant experimental tools, to make it easier for researchers to find fly strains and reagents with particular characteristics. In addition, we have added two new broad classes of experimental tool. First, Experimental Tool Reports have been generated for genetically encoded sensors, allowing researchers to find transgenic reagents that can be used to monitor changes in the levels of small molecules (e.g. GCaMP) or to monitor biophysical properties such as pH (e.g. pHluorinE) or membrane potential (e.g. Voltron). Secondly, Experimental Tool Reports have been made for reagents that can be used to modify the activity of an excitable cell; these are divided into neuron activation tools (e.g. Chrimson) and neuron inhibition tools (e.g. Kir2.1). The ‘Alleles, Insertions, and Transgenic Constructs’ tables on Gene Reports and the results of the QuickSearch ‘GAL4etc.’ tab have been modified to include experimental tool information where relevant.

### Updates to GAL4 etc QuickSearch tab

The GAL4 etc QuickSearch tab allows FlyBase users to search for transgenic drivers or reporters by temporal-spatial expression pattern, using terms from the FlyBase Developmental Ontology, the Drosophila Anatomy Ontology and the GO in defined fields. In the updated tool, users can alternatively search for drivers/reporters that reflect the expression pattern of a specific gene. Searches from the GAL4 etc QuickSearch tab now result in a responsive table-styled hitlist that includes every driver and reporter that matches the searched-for pattern and allows the user to filter by encoded tool (e.g. lexA), tool use (e.g. binary expression system - driver), tag (e.g. Tag:MYC), or tag use (e.g. epitope tag) (Figure 1B).

### Frequently Used GAL4 Drivers table

The GAL4 etc. tab also includes a link to the Frequently Used GAL4 Drivers table, a resource in which FlyBase has compiled information for more than 250 GAL4 drivers. This includes the 150 stocks most ordered from the Bloomington Drosophila Stock Center, and those drivers that have been curated to more than 20 publications. We have condensed expression information included in this resource to emphasize how drivers are being used by the research community as experimental tools. This expression information includes commonly used synonyms to controlled vocabulary anatomy terms, and brief text descriptions. We have also added representative expression pattern images to drivers where possible (Figure 1C).

## NEW REPRESENTATIONS OF FUNCTIONAL DATA

### Pathway Reports

Research in *D. melanogaster* has been central to the discovery and characterization of many important signaling pathways. To facilitate access to data on signaling pathway components, we have produced a new curated resource: Pathway Reports. To date, these encompass 16 pathways, including 6 receptor tyrosine kinase pathways and other major developmental pathways such as Hedgehog, Notch and Wnt.

**Figure 2.**
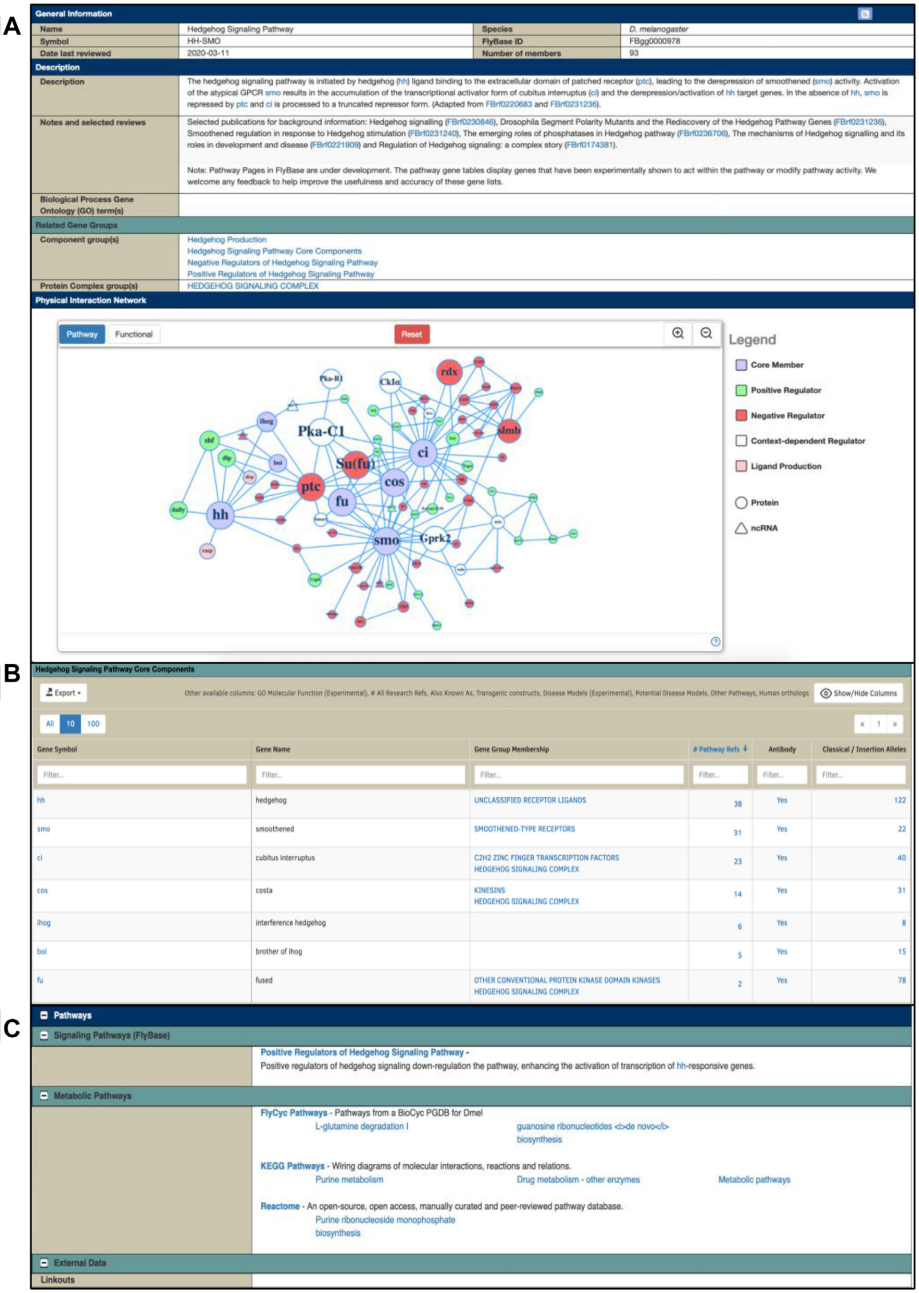
(**A**) The ‘Hedgehog Signaling Pathway’ page includes a description of the pathway, links to sub-groups and other related gene groups. The interactive Physical Interaction Network contains pathway gene members as nodes and physical interactions form the edges between them. The node size for each gene increases in the range 1–10 supporting papers, with no increase beyond 10, showing the relative experimental data for each gene's inclusion. (**B**) The members table for the Hedgehog Signaling Pathway Core Components is shown, with columns ordered by the number of references with curated supporting experimental data. (**C**) Gene Reports now feature a ‘Pathways’ section, which includes links to FlyBase Pathway pages and other pathway resources.

Pathway Reports are organized in a hierarchical fashion, with a top-level parent report and sub-groups (Figure 2A). Pathways are divided into ‘core’, ‘positive regulators’ and ‘negative regulators’ sub-groups. The ‘core’ set is defined as those genes that are required for the activated pathway to function; ‘positive regulators’ and ‘negative regulators’ act on core members of the pathway to modify their activity. A physical interaction network is also displayed for each top-level pathway group, built using Cytoscape.js (5) (Figure 2A). The network is generated by coupling Pathway Report data with our manually curated physical interaction data.

Within each Pathway Report, the member genes are listed in a customizable table (Figure 2B). With the aim of reflecting the extent to which any given pathway member's involvement is supported, we redundantly curate experimental evidence from different research papers, using defined criteria for pathway inclusion. The number of papers that provide direct experimental evidence for a gene's role in a pathway are displayed in the Members table, in the ‘# Pathway Refs’ column (Figure 2B). This information should help users differentiate between a novel regulator and a well-documented central pathway component, for instance. As part of on-going curation, additional members/supporting evidence are added to these pages to keep them current. Buttons are provided to export member genes to the ‘Batch Download’ tool or to a standard FlyBase hitlist for further refinement or analysis, or to our ortholog tool to obtain a list of predicted orthologs using data from the DRSC Integrative Ortholog Prediction Tool (DIOPT, 6).

Pathway Reports can be accessed via a dedicated ‘Pathways’ tab on the QuickSearch tool of the FlyBase homepage or from links in the ‘Function’ and ‘Pathways’ sections of the Gene Report (Figure 2C). The ‘Pathways’ section of a Gene Report also includes a ‘Metabolic Pathways’ subsection and has linkouts to other metabolic pathway resources—BioCyc (7), KEGG (8) and Reactome (9).

### Enhanced annotation and display of enzyme data

Almost a third of protein-coding genes in *D. melanogaster* encode enzymes, reflecting their critical importance for biological processes. Recently, we have made focused efforts to review the functional annotations of all enzyme-encoding genes, as well as improve the display of enzyme nomenclature and the reactions they catalyze.

We have systematically collated and reviewed *D. melanogaster* enzyme data obtained from FlyBase, UniProtKB (10) and KEGG (8), utilizing the annotations made using the GO (1) and/or Enzyme Commision (EC) classification at those resources. In addition, we generated lists of predicted enzymes based on annotations to human orthologs. This integrative approach allows for a comparative and critical assessment of existing annotation data, resulting in the addition of new enzyme annotations to FlyBase, as well as the identification and removal of incorrect annotations (11). We reviewed all the major enzyme classes, namely oxidoreductases, transferases, hydrolases, lyases, isomerases and ligases. These classes have been assembled into hierarchical Gene Group Reports at FlyBase (12). Similar to the Pathway Reports described above, these reports tabulate the members of specific enzyme groups, alongside key relevant GO terms, source references and external resources, including equivalent human enzyme groups (13).

The enzyme group to which a gene (product) belongs is shown within the ‘Function’ section of a Gene Report. This section also contains a new ‘Catalytic activity (EC)’ field, which displays the reaction(s) catalyzed by the enzyme. At the top of the Gene Report, the ‘General Information’ panel now contains an ‘Enzyme Name (EC)’ field that displays the systematic name for a given enzyme, together with its Enzyme Commission (EC) number. Showing the systematic name is especially useful where the fly gene is either unnamed or has been named based on its mutant phenotype rather than its wild-type function. EC data are derived from our GO Molecular Function annotations, taking advantage of the EC cross-references within the GO. This allows the catalytic activities to be separated into those based on experimental evidence versus those based only on computational predictions, as well as allowing automatic updates to EC data in line with GO annotation revisions.

Enzyme data within FlyBase may be queried either via GO annotations or Gene Group membership using the GO or Gene Group tabs, respectively, of the QuickSearch tool.

## UPDATES TO DISEASE MODELS

### Potential models based on orthology

**Figure 3.**
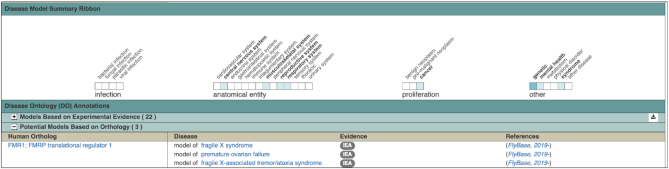
Disease model summary ribbon and ‘potential disease model’ annotations for *Fmr1*. The subsection showing potential disease models (based on orthology to human genes associated with disease ‘disease genes’), indicates that *Drosophila melanogaster Fmr1* may model three different diseases based on orthology to human FMR1. These computed disease model annotations are combined with experimental annotations (included within the closed section in this screenshot) to populate the disease summary ribbon.

We have implemented an automated pipeline to annotate *D. melanogaster* genes predicted to be relevant to disease via their orthology to human disease-associated genes. The new pipeline combines three sets of data: (i) *D. melanogaster*-to-human orthology relationships from DIOPT (6); (ii) human gene-to-phenotype relationships from OMIM (14); and (iii) OMIM phenotype cross-references within the Disease Ontology (DO) (15). The addition of orthology-based disease models complements our existing ongoing manual curation of fly models of human disease based on experimental data from published papers. The result is that both orthology-derived and experimentally based disease data in FlyBase are now indexed using terms from the DO, allowing both data sets to be queried and compared. The annotations are presented within the ‘Human Disease Associations’ section on a Gene Report, clearly separated into subsections named ‘Models Based on Experimental Evidence’ and ‘Potential Models Based on Orthology’ (Figure 3). All annotations are also included in our downloadable file of fly disease model data. At the time of implementation (FB2019_03), the new orthology-based pipeline created >3800 new DO annotations, including >3600 novel fly gene-to-disease associations.

### Disease model summary ribbons

The integration of orthology-based disease model annotations, and the consequent increase in the total number of gene-to-disease annotations, made it both feasible and useful to summarize fly disease model data in a ribbon display. This new ribbon appears above the DO annotations within the ‘Human Disease Associations’ section of Gene Reports, and shows relatively high-level DO terms separated into the broad categories of infection, anatomical entity, proliferation and other diseases (Figure 3). This ribbon is analogous to the disease model ribbon displays shown in gene pages at the Alliance of Genome Resources (16).

## UPDATES TO EXPRESSION DATA

### Expression Summary Ribbons

For an overview of expression data, we have introduced ‘Expression Summary Ribbons’ to the ‘Expression Data’ section of Gene Report pages, presenting anatomical and stage-specific expression data in a simple graphical summary. The data presented is derived from two sources: anatomy expression data manually curated by FlyBase from individual publications and high-throughput stage-specific RNA-seq data from the modENCODE project (17). The anatomy expression ribbon groups polypeptide and transcript expression data under high-level anatomy terms and presents it in a simple yes/no ribbon in which the presence of curated data is marked by a colored tile. The stage-specific RNA-seq data, which shows the variation in transcript levels throughout development, is presented as a heat map.

### Developmental Proteome

FlyBase has incorporated quantitative protein expression from the proteomic study of Casas-Vila *et al.* (18). Measurable protein expression was obtained for over half of annotated protein coding genes in one or both of two time courses: complete life cycle and embryogenesis. The expression data are presented as color-coded histograms in the ‘High-Throughput Expression Data’ sub-section of the ‘Expression Data’ section in Gene Reports. The display can be viewed at log or linear scale, with options to scale the data relative to the gene's maximum expression or relative to global ‘low’ or ‘moderate’ expression levels; the data can be downloaded to a TSV file. Together with the RNA-based FlyAtlas and modENCODE expression datasets (17,19–20), this proteome provides insights into regulation of gene expression at the level of translation and protein stability. Additionally, the peptides observed in this study have been mapped to the genome and can be viewed on JBrowse.

## MIGRATION TO THE JBrowse GENOME BROWSER

FlyBase has completed its upgrade from GBrowse to the JBrowse genome browser (4). Currently, both GBrowse and JBrowse are available on FlyBase, but GBrowse will be phased out over the next year. JBrowse offers many advantages over GBrowse including faster performance, better scaling of large datasets, new data visualization features and improved options for searching and browsing available data tracks. Additionally, we have reorganized data tracks into a new hierarchical tree with more intuitive section headings.

Recently added JBrowse tracks cover a wide range of data types. The FlyAtlas2 RNA-seq data tracks provide nucleotide-level views of transcription for various tissues, including adult male and female samples for studies of sexual dimorphism, as well as mapping of expression for microRNAs (21). As a complement to the tissue-specific RNA-seq data, the ‘SRA aggregated’ RNA-seq tracks combine data from thousands of high quality SRA RNA-seq submissions to provide an ‘average’ view of the transcriptome with exceptional read depth for high sensitivity in regions of low transcription (Brian Oliver and Justin Fear, unpublished, FBrf0241954). The ‘Polyadenylation Sites’ track offers a view of alternative transcript polyadenylation compiled from various studies (22,23). The ‘Developmental Proteome’ track displays peptides identified in Casas-Vila *et al.* (18).

A ‘DGRP Variants’ track is now available for a view of over 4 million naturally occurring variants for 205 DGRP inbred lines (24); this includes single nucleotide variants, multiple nucleotide variants, insertions and deletions. For each variant, the position and change are reported (relative to the reference genome assembly), as well as the overall frequency of the variant among the 205 strains, and the specific genotype for each of the 205 strains.

FlyBase now offers a set of tracks to support the use or design of short guide RNAs (sgRNAs). sgRNAs that have been incorporated into transgenic lines for expression in flies can be browsed by collection (25–27) and there is also a set of sgRNA prediction tracks that assess over 10 million possible sgRNA designs for possible off-target effects and predicted efficacy (28,29).

## PARALOGS

**Figure 4.**
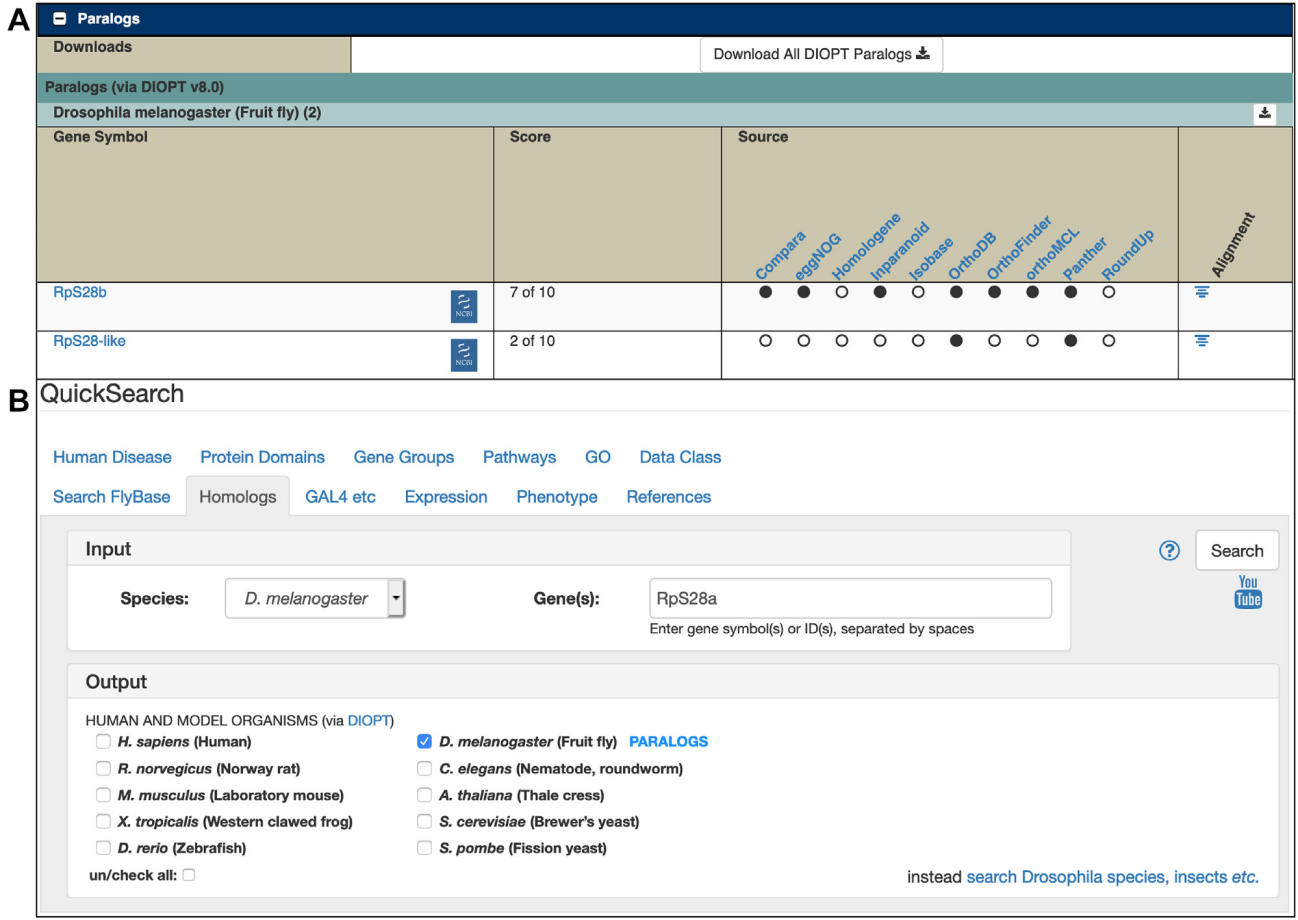
(**A**) Paralogs section of the *Drosophila melanogaster RpS28a* report, showing that two other genes (*RpS28b* and *RpS28-like*) are identified as paralogs by the indicated sources. A link is provided to ‘Download all DIOPT Paralogs’, which is useful if many paralogs are listed. (**B**) Paralogs can be searched using the ‘Homologs’ tab of the QuickSearch tool by selecting ‘*D. melanogaster*’ as both the input and output species.

A substantial number of genes are duplicated in the *D. melanogaster* genome (30,31). This can result in genetic redundancy and is particularly relevant for RNAi-based experiments. We have recently incorporated paralog predictions from the DIOPT (6), which integrates pairwise paralog calls from 10 individual algorithms. Significantly, nearly half (46%) of all *D. melanogaster* genes are predicted to have at least one paralog by two or more algorithms. Paralogy data are presented within a dedicated ‘Paralogs’ section of each Gene Report (Figure 4A) and are searchable (alongside orthology data) within the renamed ‘Homologs’ tab of QuickSearch on the FlyBase homepage (Figure 4B). A new downloadable file containing all *D. melanogaster* paralog data is also available via the ‘Downloads’ menu in the navigation bar of any FlyBase page.

## IMPROVEMENTS TO BATCH QUERIES AND DOWNLOADS

**Figure 5.**
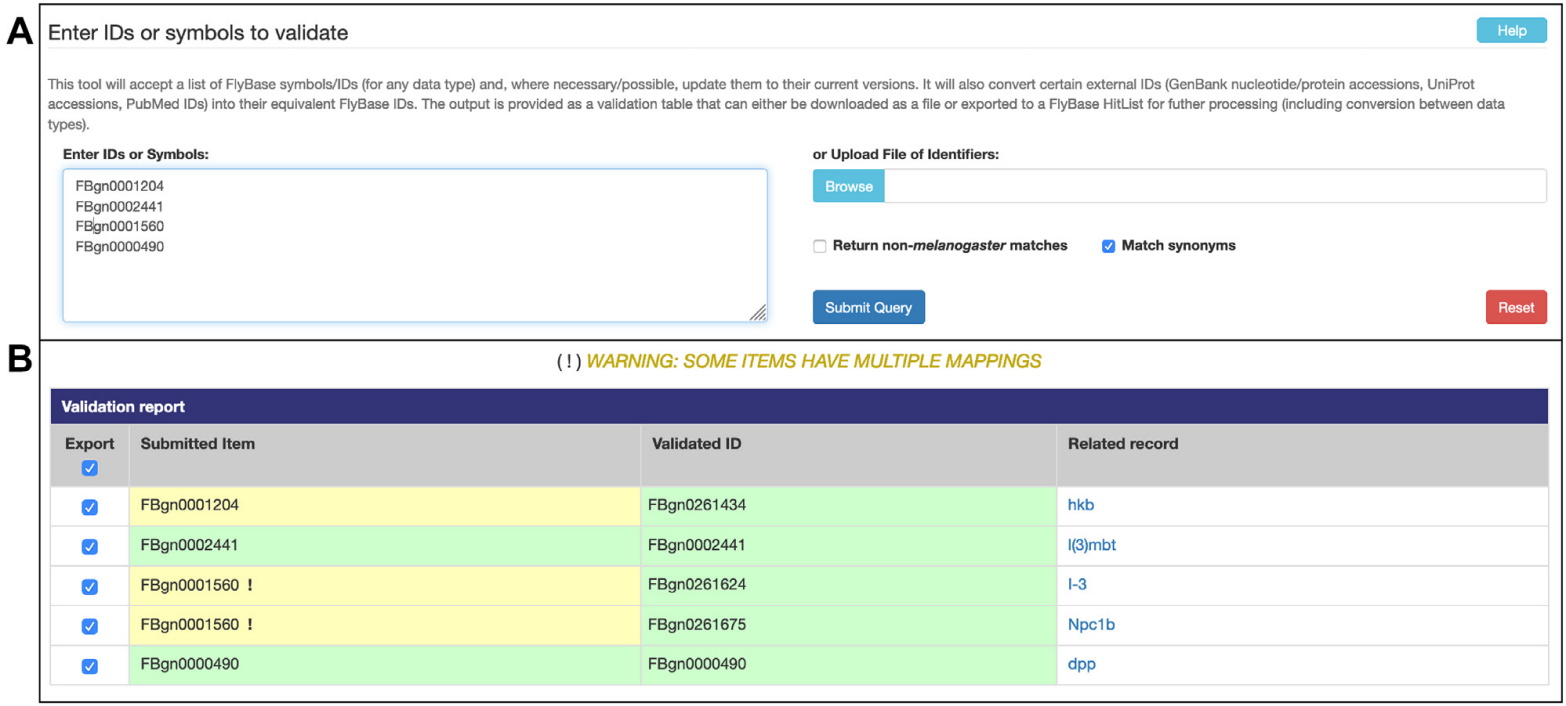
ID Validator input form (**A**) and output page (**B**). Submitted items that have been updated from their original values are highlighted with a pale yellow background. In cases where a single submitted item is now represented by two or more validated IDs, an exclamation mark ‘!’ is appended to the value in the ‘Submitted Item’ column.

We have made several improvements to help users perform batch queries/downloads of FlyBase data. Our ID Validator (previously ‘ID converter’) tool accepts a list of FlyBase symbols/IDs and, where necessary/possible, updates them to their current versions. It will also convert certain external IDs (GenBank nucleotide/protein accessions, UniProt accessions, PubMed IDs) into their equivalent FlyBase IDs. The output is provided as a validation table that can either be downloaded as a file, or exported to a FlyBase hitlist for further processing or to Batch Download to obtain associated data. In addition to a performance fix to handle large lists of items, ID Validator has been enhanced with several new features: *D. melanogaster* IDs are now the default, with the option to include other species if desired; the ability to enable/disable the use of symbol synonyms; highlighting of updated IDs requiring manual review; and the ability to select items for export (Figure 5).

ID Validator may be accessed via the ‘Tools → Query by symbols/IDs’ menu in the navigation bar present on all FlyBase pages. We have also added a prominent link to ID Validator to the top of our Batch Download tool. This tool accepts a list of IDs/symbols and retrieves user-specified associated data from report pages or precomputed files, but it will only generate complete and accurate results if the submitted IDs/symbols are current and valid.

Many precomputed bulk data files are generated with each FlyBase release and can be accessed via the ‘Downloads’ menu in the navigation bar or directly via our FTP site (ftp://ftp.flybase.org/releases/). A recent survey demonstrated that our precomputed files were valued and well used by our community, and led to several improvements in their provision. These included adding help links/README files to the web page listing of files/FTP folder, respectively, and the inclusion of additional precomputed files as data sources within the Batch Download interface.

We have also released a dedicated ‘FlyBase for Developers’ site (https://flybase.github.io), which provides documentation for those wishing to learn more about the various ways of accessing FlyBase data. The site currently includes API docs, HowTos, information on how to query and access Chado (our relational database), and bulk download files.

## COMMUNITY INTERACTIONS

### FlyBase Twitter

FlyBase maintains an active Twitter profile (https://twitter.com/FlyBaseDotOrg) that we use to alert users to new data and features, and to share topical news relevant to the fly community. We also post ‘tweetorials’, a short series of linked tweets that visually guide users through the details of new or improved FlyBase features and tools.

### Improvements to the Fast-Track Your Paper tool

The Fast-Track Your Paper tool was first introduced to FlyBase in 2009 and has undergone a number of upgrades (32). The tool allows first-pass curation of publications by authors, indicating to curators the types of data in the paper and also resulting in relevant genes being associated with the reference. This tool benefits FlyBase as it facilitates curation of these papers, and authors benefit as incorporation of their data into FlyBase is accelerated. We have recently made several improvements to the tool, in order to enhance usability and give users options to submit more information about their paper. The Fast-Track Your Paper tool may be accessed via the ‘Tools’ menu in the navigation bar present on all FlyBase pages. However, most authors will interact with the tool in response to an email from FlyBase to the corresponding author once their publication has been fully published (i.e. has final volume and page numbers) and added to the FlyBase bibliography.

Our reference search within the tool now shows if a particular reference has already been curated, to reduce duplicated effort by authors. Reviews are now included in the search, and review authors will also be contacted when their publication is added to FlyBase. This will allow authors to quickly associate genes to their reference using a slimmed down version of the tool, giving users better access to reviews relevant to particular genes. We have refined and updated the ‘data types’ step, allowing authors to indicate more accurately which kinds of data are included in their publication and optionally contribute more detail where appropriate. To aid authors, we now also pre-populate four data types (disease, new allele, new transgene, physical interaction) if they have been detected by our text-mining pipeline (33,34). The ‘associate genes’ step in the tool has also been enhanced to allow searching of genes via synonyms and facilitate finding human genes that have been introduced into *D. melanogaster*. If a user selects the option to bulk upload genes, we encourage them to use the new ‘dataset’ flag if there is a large number of genes, and provide a link to the upgraded ‘ID validator’ tool so that correct current gene symbols can be used.

## FLYBASE AS A HUB: LINKS TO/FROM OTHER DATABASES

For many years, FlyBase has provided/generated custom data files for ingest by other biological databases. Notable among these are NCBI GenBank/RefSeq (35, annual update to *D. melanogaster* genome annotations), UniProtKB (10, cross references linking current FlyBase protein-coding gene IDs to SwissProt/TrEMBL protein accessions), RNAcentral (36, FlyBase IDs, classifications and genome annotation data for non-coding genes/transcripts), the HGNC (13, FlyBase Gene Group data) and the Alliance of Genome Resources (16, see below). Likewise, FlyBase imports/updates data from these and many other sources, either for each two-monthly FlyBase release or annually. This is important from a user perspective, as it enables current reciprocal links to be made between related databases.

The Alliance of Genome Resources is a consortium of six major model organism databases and the GO, with the aim of facilitating the exploration of related genes in human and well-studied model organisms (16). As a member of this consortium, FlyBase exports all relevant data to the Alliance central database.

On the FlyBase website, links to external resources are consolidated near the foot of each report page in the section named ‘External Crossreferences and Linkouts’. We have also recently added a ‘Key Links’ section to the top of Gene Reports, which provides quick prominent links to the Alliance of Genome Resources (16), NCBI Gene (35), Ensembl (37), and UniProtKB (10) or RNAcentral (36).

## GOING FORWARD

Since its establishment in 1992, FlyBase has facilitated access to a wide variety of up-to-date *D. melanogaster* data, along with provision of an assortment of tools to facilitate biological discovery. As FlyBase tries to keep pace with new developments in Drosophila research, tasks are re-evaluated in terms of their cost and relative benefit to our community. With this in mind, FlyBase no longer maintains updated gene model annotations or genome assembly information for non-*D. melanogaster* Drosophila species. Going forward, this will allow FlyBase to focus on providing high quality *D. melanogaster* information that is critical to the community.

## References

[1] The Gene Ontology Consortium. The Gene Ontology Resource: 20 years and still GOing strong. Nucleic Acids Res. 2019; 47:D330–D338. 30395331 10.1093/nar/gky1055 PMC6323945

[2] Marygold S.J., Crosby M.A., Goodman J.L. The FlyBase Consortium. Using FlyBase, a database of *Drosophila* genes and genomes. Methods Mol. Biol. 2016; 1478:1–31. 27730573 10.1007/978-1-4939-6371-3_1 PMC5107610

[3] Thurmond J., Goodman J.L., Strelets V.B., Attrill H., Gramates L.S., Marygold S.M., Matthews B.M., Millburn G., Antonazzo G., Trovisco V. et al. FlyBase 2.0: the next generation. Nucleic Acids Res. 2019; 47:D759–D765. 30364959 10.1093/nar/gky1003 PMC6323960

[4] Buels R., Yao E., Diesh C.M., Hayes R.D., Munoz-Torres M., Helt G., Goodstein D.M., Elsik C.G., Lewis S.E., Stein L. et al. JBrowse: a dynamic web platform for genome visualization and analysis. Genome Biol. 2016; 17:66. 27072794 10.1186/s13059-016-0924-1 PMC4830012

[5] Franz M., Lopes C.T., Huck G., Dong Y., Sumer O., Bader G.D. Cytoscape.js: a graph theory library for visualisation and analysis. Bioinformatics. 2016; 32:309–311. 26415722 10.1093/bioinformatics/btv557 PMC4708103

[6] Hu Y., Flockhart I., Vinayagam A., Bergwitz C., Berger B., Perrimon N., Mohr S.E. An integrative approach to ortholog prediction for disease-focused and other functional studies. BMC Bioinformatics. 2011; 12:357. 21880147 10.1186/1471-2105-12-357 PMC3179972

[7] Karp P.D., Billington R., Caspi R., Fulcher C.A., Latendresse M., Kothari A., Keseler I.M., Krummenacker M., Midford P.E., Ong Q. et al. The BioCyc collection of microbial genomes and metabolic pathways. Brief. Bioinform. 2019; 20:1085–1093. 29447345 10.1093/bib/bbx085 PMC6781571

[8] Kanehisa M., Furumichi M., Tanabe M., Sato Y., Morishima K. KEGG: new perspectives on genomes, pathways, diseases and drugs. Nucleic Acids Res. 2017; 45:D353–D361. 27899662 10.1093/nar/gkw1092 PMC5210567

[9] Jassal B., Matthews L., Viteri G., Gong C., Lorente P., Fabregat A., Sidiropoulos K., Cook J., Gillespie M., Haw R. et al. The reactome pathway knowledgebase. Nucleic Acids Res. 2020; 48:D498–D503. 31691815 10.1093/nar/gkz1031 PMC7145712

[10] The UniProt Consortium. UniProt: a worldwide hub of protein knowledge. Nucleic Acids Res. 2019; 47:D506–D515. 30395287 10.1093/nar/gky1049 PMC6323992

[11] Garapati P.V., Zhang J., Rey A.J., Marygold S.J. Towards comprehensive annotation of Drosophila melanogaster enzymes in FlyBase. Database (Oxford). 2019; 2019:bay144. 10.1093/database/bay144 PMC6343044 30689844

[12] Attrill H., Falls K., Goodman J.L., Millburn G.H., Antonazzo G., Rey A.J., Marygold S.J. the FlyBase Consortium. FlyBase: establishing a Gene Group resource for Drosophila melanogaster. Nucleic Acids Res. 2016; 44:D786–D792. 26467478 10.1093/nar/gkv1046 PMC4702782

[13] Braschi B., Denny P., Gray K., Jones T., Seal R., Tweedie S., Yates B., Bruford E. Genenames.org: the HGNC and VGNC resources in 2019. Nucleic Acids Res. 2019; 47:D786–D792. 30304474 10.1093/nar/gky930 PMC6324057

[14] Amberger J.S., Bocchini C.A., Scott A.F., Hamosh A. OMIM.org: leveraging knowledge across phenotype-gene relationships. Nucleic Acids Res. 2019; 47:D1038–D1043. 30445645 10.1093/nar/gky1151 PMC6323937

[15] Schriml L.M., Mitraka E., Munro J., Tauber B., Schor M., Nickle L., Felix V., Jeng L., Bearer C., Lichenstein R. et al. Human Disease Ontology 2018 update: classification, content and workflow expansion. Nucleic Acids Res. 2019; 47:D955–D962. 30407550 10.1093/nar/gky1032 PMC6323977

[16] The Alliance of Genome Resources Consortium. Alliance of Genome Resources Portal: unified model organism research platform. Nucleic Acids Res. 2020; 48:D650–D658. 31552413 10.1093/nar/gkz813 PMC6943066

[17] Graveley B.R., Brooks A.N., Carlson J.W., Duff M.O., Landolin J.M., Yang L., Artieri C.G., van Baren M.J., Boley N., Booth B.W. et al. The developmental transcriptome of Drosophila melanogaster. Nature. 2011; 471:473–479. 21179090 10.1038/nature09715 PMC3075879

[18] Casas-Vila N., Bluhm A., Sayols S., Dinges N., Dejung M., Altenhein T., Kappei D., Altenhein B., Roignant J., Butter F. The developmental proteome of *Drosophila melanogaster*. Genome Res. 2017; 27:1273–1285. 28381612 10.1101/gr.213694.116 PMC5495078

[19] Chintapalli V.R., Wang J., Dow J.A. Using FlyAtlas to identify better Drosophila melanogaster models of human disease. Nat. Genet. 2007; 39:715–720. 17534367 10.1038/ng2049

[20] Brown J.B., Boley N., Eisman R., May G.E., Stoiber M.H., Duff M.O., Booth B.W., Wen J., Park S., Suzuki A.M. et al. Diversity and dynamics of the Drosophila transcriptome. Nature. 2014; 512:393–399. 24670639 10.1038/nature12962 PMC4152413

[21] Leader D.P., Krause S.A., Pandit A., Davies S.A., Dow J.A.T. FlyAtlas 2: a new version of the Drosophila melanogaster expression atlas with RNA-Seq, miRNA-Seq and sex-specific data. Nucleic Acids Res. 2018; 46:D809–D815. 29069479 10.1093/nar/gkx976 PMC5753349

[22] Liu X., Freitas J., Zheng D., Oliveira M.S., Hoque M., Martins T., Henriques T., Tian B., Moreira A. Transcription elongation rate has a tissue-specific impact on alternative cleavage and polyadenylation in *Drosophila melanogaster*. RNA. 2017; 23:1807–1816. 28851752 10.1261/rna.062661.117 PMC5689002

[23] Sanfilippo P., Wen J., Lai E.C. Landscape and evolution of tissue-specific alternative polyadenylation across Drosophila species. Genome Biol. 2017; 18:229. 29191225 10.1186/s13059-017-1358-0 PMC5707805

[24] Huang W., Massouras A., Inoue Y., Peiffer J., Ràmia M., Tarone A.M., Turlapati L., Zichner T., Zhu D., Lyman R.F. et al. Natural variation in genome architecture among 205 Drosophila melanogaster Genetic Reference Panel lines. Genome Res. 2014; 24:1193–1208. 24714809 10.1101/gr.171546.113 PMC4079974

[25] Zirin J., Hu Y., Liu L., Yang-Zhou D., Colbeth R., Yan D., Ewen-Campen B., Tao R., Vogt E., VanNest S. et al. Large-scale transgenic *Drosophila* resource collections for loss- and gain-of-function studies. Genetics. 2020; 214:755–767. 32071193 10.1534/genetics.119.302964 PMC7153935

[26] Meltzer H., Marom E., Alyagor I., Mayseless O., Berkun V., Segal-Gilboa N., Unger T., Luginbuhl D., Schuldiner O. Tissue-specific (ts)CRISPR as an efficient strategy for in vivo screening in Drosophila. Nat. Commun. 2019; 10:2113. 31068592 10.1038/s41467-019-10140-0 PMC6506539

[27] Port F., Strein C., Stricker M., Rauscher B., Heigwer F., Zhou J., Beyersdörffer C., Frei J., Hess A., Kern K. et al. Tissue-specific (ts)CRISPR as an efficient strategy for in vivo screening in Drosophila. Elife. 2020; 9:e53865. 32053108 10.7554/eLife.53865 PMC7062466

[28] Ren X., Sun J., Housden B.E., Hu Y., Roesel C., Lin S., Liu L.P., Yang Z., Mao D., Sun L. et al. Optimized gene editing technology for Drosophila melanogaster using germ line-specific Cas9. Proc. Natl. Acad. Sci. U.S.A. 2013; 110:19012–19017. 24191015 10.1073/pnas.1318481110 PMC3839733

[29] Housden B.E., Valvezan A.J., Kelley C., Sopko R., Hu Y., Roesel C., Lin S., Buckner M., Tao R., Yilmazel B. et al. Identification of potential drug targets for tuberous sclerosis complex by synthetic screens combining CRISPR-based knockouts with RNAi. Sci. Signal. 2015; 8:rs9. 26350902 10.1126/scisignal.aab3729 PMC4642709

[30] Heger A., Ponting C.P. Evolutionary rate analyses of orthologs and paralogs from 12 Drosophila genomes. Genome Res. 2007; 17:1837–1849. 17989258 10.1101/gr.6249707 PMC2099592

[31] Bao R., Dia S.E., Issa H.A., Alhusein D., Friedrich M. Comparative evidence of an exceptional impact of gene duplication on the developmental evolution of Drosophila and the Higher Diptera. Front. Ecol. Evol. 2018; 6:63.

[32] Bunt S.M., Grumbling G.B., Field H.I., Marigold S.J., Brown N.H., Millburn G.H. the FlyBase Consortium. Directly e-mailing authors of newly published papers encourages community curation. Database (Oxford). 2012; 2012:bas024. 22554788 10.1093/database/bas024 PMC3342516

[33] McQuilton P. the FlyBase Consortium. Opportunities for text mining in the FlyBase genetic literature curation workflow. Database (Oxford). 2012; 2012:bas039. 23160412 10.1093/database/bas039 PMC3500518

[34] Fang R., Schindelman G., Van Auken K., Fernandes J., Chen W., Wang X., Davis P., Tuli M.A., Marygold S., Millburn G. et al. Automatic categorization of diverse experimental information in the bioscience literature. BMC Bioinformatics. 2012; 13:16. 22280404 10.1186/1471-2105-13-16 PMC3305665

[35] NCBI Resource Coordinators. Database resources of the National Center for Biotechnology Information. Nucleic Acids Res. 2018; 46:D8–D13. 29140470 10.1093/nar/gkx1095 PMC5753372

[36] The RNAcentral Consortium RNAcentral: a hub of information for non-coding RNA sequences. Nucleic Acids Res. 2019; 47:D221–D229. 30395267 10.1093/nar/gky1034 PMC6324050

[37] Yates A.D., Achuthan P., Akanni W., Allen J., Alvarez-Jarreta J., Amode M.R., Armean I.M., Azov A.G., Bennett R., Bhai J. et al. Ensembl 2020. Nucleic Acids Res. 2020; 48:D682–D688. 31691826 10.1093/nar/gkz966 PMC7145704

